# A multicenter, randomized, open-label, active-comparator trial to determine the efficacy, safety, and pharmacokinetics of intravenous ibuprofen for treatment of fever in hospitalized pediatric patients

**DOI:** 10.1186/s12887-017-0795-y

**Published:** 2017-02-01

**Authors:** Samia N. Khalil, Barry J. Hahn, Corrie E. Chumpitazi, Amy D. Rock, Byron A. Kaelin, Charles G. Macias

**Affiliations:** 1 Department of Anesthesiology, The University of Texas Medical School at Houston, 6431 Fannin Street, MSB 5.020, Houston, TX 77030 USA; 2 Department of Emergency Medicine, Staten Island University Hospital, 475 Seaview Avenue, Staten Island, New York, 10305 USA; 3 Department of Pediatrics, Baylor College of Medicine, Texas Children’s Hospital, 6621 Fannin Street, Suite A2210, Houston, TX 77030 USA; 4 Cumberland Pharmaceuticals Inc., 2525 West End Avenue, Suite 950, Nashville, TN 37203 USA

**Keywords:** Antipyretic, Fever, Ibuprofen, NSAIDs, Pediatric

## Abstract

**Background:**

Oral antipyretics are commonly used to treat pediatric patients who develop fevers. However, patients presenting to the emergency department or undergoing surgery are frequently unable to tolerate oral antipyretics. Rectal formulations are available; however, this route of administration is unpredictable. The main objectives of this randomized controlled study was to evaluate the efficacy and safety of single or multiple doses of intravenous ibuprofen to acetaminophen (oral or suppository) in pediatric patients with fever and to assess plasma ibuprofen concentrations.

**Methods:**

This multi-center study was conducted in hospitalized patients, ≤ 16 years, with a new onset of fever ≥ 38.3°C. Patients were randomly assigned to receive either 10 mg/kg intravenous ibuprofen or acetaminophen. Study drug was administered at hour 0, and thereafter every 4 h as needed, up to 5 days. The primary outcome was to evaluate the effect of a single dose of intravenous ibuprofen compared to acetaminophen in reducing temperature in the first 2 h after administration. Data were compared using an analysis of variance model for continuous measurements and Cochran-Mantel-Haenszel test of general association for categorical data. A two-sided testing was used and a *p*-value ≤ 0.05 was considered significant.

**Results:**

A total of 103 patients received study medication. Intravenous ibuprofen resulted in a greater reduction in temperature as measured by the area under the change from baseline at 2 h (*p* = 0.005) and 4 h (<0.001); in a greater reduction in change from baseline temperature compared to treatment with acetaminophen, and it reduced fever throughout a 24 h dosing period. There were no differences in safety parameters or serious adverse events.

**Conclusions:**

A single 10 mg/kg dose of intravenous ibuprofen provided a significant reduction of temperature for febrile pediatric patients compared to those that received 10 mg/kg acetaminophen at 2 h and 4 h post-treatment. A reduction in temperature was also demonstrated over 24 h; however the reduction was not considered statically significant. Intravenous ibuprofen provides an effective option for reducing fever in hospitalized pediatric patients.

**Trial registration:**

The study was registered on ClinicalTrials.gov on 26 October 2009, Study Identifier: NCT01002573

**Electronic supplementary material:**

The online version of this article (doi:10.1186/s12887-017-0795-y) contains supplementary material, which is available to authorized users.

## Background

Fever is one of the common symptoms managed by health care providers and one of the leading reasons children and infants present for medical evaluation, accounting for nearly 65% ambulatory pediatric visits and frequently leading to the administration of antipyretic medications [1–3]. Indications for initiating antipyretic therapy are a temperature higher than 38.3° Celsius (C) [101°Fahrenheit (F)] and improving the child’s overall comfort [3]. Ibuprofen is an anti-pyretic and analgesic recommended worldwide as a first-line agent for the treatment of pain and fever in adults and children. In double blind trials it provides significantly better fever reduction than placebo and has long been available in oral solid and liquid forms.

Oral antipyretics are commonly used in hospitals to treat pediatric patients who develop fevers. However, patients presenting to the emergency department, undergoing surgery, or those admitted to the hospital are frequently unable to ingest, digest, absorb, or tolerate oral antipyretics. Rectal formulations are available for some medications, such as acetaminophen; however, this route of administration produces peak levels that may vary by as much as nine-fold and often will not achieve therapeutic levels after recommended doses are administered [4]. During study design and upon study initiation, intravenous (IV) antipyretics, including IV acetaminophen were not Food and Drug Administration (FDA) approved and commercially available in the United States; therefore, to design a comparator study, an oral acetaminophen comparator arm was included.

An intravenous form of ibuprofen was approved by the FDA in 2009 for the treatment of pain and fever in adults under the brand name Caldolor^®^ (Ibuprofen Injection, Cumberland Pharmaceuticals Inc., Nashville, TN) [5]. IV ibuprofen has been shown to be successful in the treatment of pain and fever in adults [6–10]. Also, a single preoperative dose of 10 milligrams/kilogram (mg/kg) intravenous ibuprofen was found to significantly reduce fentanyl use in pediatric tonsillectomy patients, in a multicenter, randomized, double-blind placebo-controlled study [11]. This study was conducted to provide data in support of intravenous ibuprofen in hospitalized febrile pediatric patients.

This multicenter, open-label study was designed to evaluate the efficacy and safety of a single dose of intravenous ibuprofen compared to acetaminophen (oral or suppository) in pediatric patients with fever. Additional objectives included the evaluation of single and multiple doses in terms of safety and efficacy. Blood samples for analysis of pharmacokinetic properties were collected to assess plasma ibuprofen concentrations.

## Methods

Study approval was granted from the Institution Review Board (IRB) of record for each clinical site. The study was conducted under an Investigational New Drug application and performed in accordance with the Declaration of Helsinki. The study was registered at ClinicalTrials.gov (Study Identifier: NCT01002573) on October 26^th^, 2006; prior to enrollment of the first subject. An independent Data and Safety Monitor (DSM) monitored study progress and outcomes.

This multi-center, randomized, open-label parallel group, active comparator study compared intravenous ibuprofen (10 mg/kg) and acetaminophen (10 mg/kg). The study was conducted in hospitalized pediatric patients sixteen (16) years of age or younger with a fever greater than or equal to 101.0° F (38.3° C) across fourteen sites within the United States (Table 1).

**Table 1:** Clinical centers enrolling patients

Investigator	Hospital
Antonia C. Arrieta, MD	Children’s Hospital of Orange County
Roger P. Barton, MD	Children’s Hospital at St. Francis
Keith A. Candiotti, MD	Jackson Memorial Hospital, University of Miami
Corrie E. Chumpitazi MD	Texas Children’s Hospital
Jeff A. Clark, MD	Children’s Hospital at Michigan
Steven A. Conrad, MD, PhD	Louisiana State University Health Science Center, Shreveport
Barry Hahn, MD	Staten Island University Hospital
James Hanley, III, MD	Ochsner Clinic Foundation
Samia Khalil, MD	UTMS-Houston, Children’s Memorial Hermann Hospital
Charles G. Macias, MD	Texas Children’s Hospital
Onyinye C. Onyekwere, MD	Howard University Hospital
David I. Rosenberg, MD	Maricopa Integrated Health System, Maricopa Medical Center
Janice E. Sullivan, MD	Kosair Children’s Hospital, University of Louisville
Cynthia Tinsley, MD	Loma Linda University Children’s Hospital
John Zhong, MD	Children’s Medical Center, Dallas

Between August 16, 2010 and April 23, 2013, written informed consent from the patient’s parent or legal guardian was obtained prior to any study-specific interventions. When appropriate, the patient, based on each site’s IRB’s assessment of risk and potential benefit, was given an age appropriate explanation of the purpose and evaluations of the study and assent was obtained.

The study included a screening/baseline period, a treatment period and a post-treatment period. Patients were eligible for the study if their age was between birth (28 weeks gestational age) to less than or equal to 16 years of age and had new onset of fever (less than 7 days) documented by temperature greater than or equal to 101.0° F (38.3° C) measured by tympanic route. Braun ThermoScan^®^ PRO 4000 Tympanic Thermometers were provided to each clinical site and tympanic measurements were required for all temperature assessments. Patients were excluded if they had one or more of the following: inadequate intravenous access; received any other antipyretic drug therapy within 2 h of dosing; history of allergy and/or hypersensitivity to any component of IV-ibuprofen (or related products); received another investigational drug within the past 30 days; fever due to malignant hyperthermia; or nursing or pregnant as determined by positive serum or urine test.

Eligible patients were randomized in a 1:1 ratio to receive either 10 mg/kg of body weight – intravenous ibuprofen (not to exceed 400 mg per dose or daily maximum dose of 2,400 mg) or 10 mg/kg of body weight - oral acetaminophen solution or equivalent rectal suppository dose (not to exceed 650 mg per dose or daily maximum dose of 3,900 mg). Subject randomization was performed by a member of the site research team utilizing an interactive web response system (TrialsSTAT, Jubliant Clinsys). Randomization was stratified according to age group (birth to < 6 months of age and 6 months to ≤ 16 years of age). After randomization, the patient’s tympanic temperature was assessed again just prior to administration of the first study drug dose. If the patient became afebrile in the duration between randomization and the start of dosing, the site was instructed not to administer the dose until the patient’s temperature was 38.3 ° C (101.0° F) or greater, and the patient met the eligibility criteria for enrollment. At baseline, demographic data and the medical history of the subject was obtained. A standard physical examination was conducted and vital signs, including temperature were measured. If the subject was of child-bearing age, pregnancy testing was performed. Adverse event data, including description, intensity, severity, and relationship to the investigational medicinal product were recorded.

Study drug at the appropriate dose was administered at hour 0, and thereafter every 4 h as needed (PRN) until afebrile or 120 h maximum. Intravenous ibuprofen was administered at a dose of 10 mg/kg of body weight over ten minutes. After the first dose of study drug was administered, tympanic route temperature was measured at 15 min intervals up to 105 min, followed by additional tympanic temperatures at hours 2, 2.5, 3, 3.5, 4, and 6 h. Additional temperature measurements were taken pre-dose, 30, 60, 120, and 240 min after any PRN dosing. Vitals signs were taken at hours 0, 1, 2, 4, 8, and 24; then prior to the start of, 30, 60, 120, and 240 min after any PRN dosing. In the ibuprofen treatment group, 2 mL blood samples were drawn in K2-EDTA tubes for pharmacokinetic profiling of ibuprofen immediately post-dose, at 30 min, 1 h, 2 h and 4 h post the initial dose. Sparse sampling for pharmacokinetic was performed for patients under 6 months of age. A total of 213 blood samples were drawn during the study for drug analysis. Plasma samples were separated by refrigerated centrifuge and then frozen at -20°C, and kept frozen until analyzed. Ibuprofen and its internal standard ibuprofen-d_3_ were extracted from 0.025mL aliquots of plasma. The extracted samples were injected into a liquid chromatograph and separated using a gradient at 25°C at a flow rate of 1.000 ml/min. The detection was made with a tandem mass spectrometry detector API 4000.

Blood samples for safety monitoring were taken at screening then at days 1 and 5 (or end of study if less than 120 h). Concomitant medications, procedures and safety monitoring were assessed and recorded continuously throughout the study for each patient. Temperature and vital signs were monitored post-treatment until discharge, at which time discharge measurements were assessed. To provide additional safety monitoring, the patient’s parent or legal guardian was contacted by telephone to obtain any adverse event information occurring through day 6.

The primary outcome of this study was to evaluate the effect of a single dose of IV ibuprofen compared to a single dose of acetaminophen in reducing temperature in the first 2 h after administration. Secondary outcomes included evaluation of the change in temperature after the first 30, 60, and 240 min of treatment, evaluation of the change in temperature versus time after 4 and 24 h of treatment, evaluation of the time to afebrile (temperature less than 100.4 °F [38 °C]) status, and to determine the percentage of patients becoming afebrile after 4 h. Additionally, secondary endpoints included the evaluation of safety and tolerability of single and repeated doses of intravenous ibuprofen by assessing adverse events (AEs), vital sign changes, and laboratory abnormalities.

### Statistical analysis

The primary endpoint of the study evaluated fever reduction within the first 120 min of treatment from baseline. The temperature immediately before dosing was considered the baseline temperature. To evaluate the primary endpoint of AUC_0-2_, the area under the change in temperature from baseline verse time curve was calculated using a linear trapezoidal rule on all available change from baseline in temperature data to two hours post-dose for each patient. If any intermediate temperature values were missing during the period, linear interpolation between the adjacent values were used as imputed values. The primary efficacy variable was evaluated between the two treatment groups using an analysis of variance (ANOVA) model with an effect for treatment group. The nonparametric method of Wilcoxon rank-sum test was also performed. If the endpoint was not normally distributed (i.e., if the Kolmogorov-Smirnov *p*-value is greater than 0.05), the results from the non-parametric method were used.

The secondary outcomes of change in temperature after the first 30 min, 60 min, and 240 min of treatment were evaluated using an ANCOVA model with effects for baseline temperature and treatment group. Missing temperature values were imputed using linear interpolation of adjacent values. If adjacent values were not available then the missing temperature values were missing for the analysis.

The secondary outcomes evaluate the change in temperature versus time after 4 h and 24 h of treatment were compared between the two treatment groups using an ANOVA model with an effect for treatment group. AUC_0_-t was derived by calculating the area under the change in temperature from baseline versus time curve using the linear trapezoidal rule on all available change from baseline in temperature data from baseline to t hours post-dose for each patient. If any intermediate temperature values were missing during the period, linear interpolation between the adjacent values were used as imputed values.

The secondary outcome of time to afebrile (temperature less than 100.4 °F [38 °C]) was analyzed by using the log-rank test and the percentage of patients becoming afebrile after 4 h was analyzed for differences between treatment groups with the Cochran-Mantel-Haenszel test of general association.

The populations of interest included in the analysis included the intent-to-treat population (ITT) which included all subjects who were randomized and received at least one partial dose of clinical trial material (CTM); the Efficacy-Evaluable Population (EEP) which included all subjects who had no major protocol violations with regard to inclusion or exclusion criteria or study conduct and had all primary efficacy assessments at baseline and at least three of the following time points: 15 min, 30 min, 45 min, 60 min, 75 min, 90 min, 105 min, or 120 min; and the Safety Population, which consisted of all subjects who were randomized and received at least a partial dose of CTM; and the pharmacokinetic (PK) population which included every subject for whom sufficient data were collected to calculate the PK parameters. PK parameters were estimated with standard non-compartmental methods using WinNonlin® version 5.3 software. Plasma concentration values from ibuprofen were used to calculate the following parameters:

- AUC_0-_t: Area under the concentration-time curve from time zero to the last measurable concentration using linear-log trapezoidal rule.
- AUC_0–4_: Area under the concentration-time curve from time zero to 4 h.
- Cmax: Maximum observed concentration.
- Tmax: Time of observed Cmax.
- T½ el: Elimination half-life, calculated as ln(2)/Kel
- Cl: Total body clearance, calculated as Dose/AUC0-inf.
- Vz: Volume of distribution, calculated as Dose/(Kel x AUC0-inf).

The terminal rate constant was calculated for all subject when possible. The value of the constant (Kel) was determined by the slope of the regression line of ln-transformed concentration vs. time profile with the following constraints: at least four non zero observations during the terminal elimination phase were used to calculate the Kel. A minimum of three observations was used if fewer than four observations were available. If the constant (Kel) could not be measured (e.g.: fewer than three non zero concentrations in the terminal elimination phase) or the determination coefficient (*r*2 value) from the regression of the ln linear elimination phase was less than 64% (or 0.64) for some patients (or *r* value positive or less than 80% or 0.80 in absolute value), then the parameters related to the elimination were not calculated for that individual pharmacokinetic profile.

Data were compared using an analysis of variance model for continuous measurements and Cochran-Mantel-Haenszel test of general association for categorical data. Data were analyzed using descriptive statistics and reported as either mean, standard deviation or standard error, median, minimum and maximum and number of patients in each treatment group. Statistical computations were performed and data appendices created using SAS^®^ system version 9.1.3 or higher. Statistical significance was defined as *p* ≤ to 0.05.

Plasma samples were analyzed for ibuprofen by the Bioanalytical division of inVentiv Health Clinical. The plasma concentrations vs time analysis was the responsibility of inVentiv Health Clinical. The PK analyses were based on the PK population. For the purpose of calculations, plasma concentrations recorded as below the limit of quantification were entered as zero up to the first detectable concentration and as missing thereafter. Actual sample collection times were used in calculations rather than scheduled collection times, except for pre-dose samples, which were always reported as zero (0.000), regardless of the time difference.

A two compartment model with proportional error provided the best fitting for ibuprofen PK. The best model included weight (WT) as a power covariate on clearances (Cl, Q) to the power ¾, and on volumes of distribution (V1, V2) to the power of 1, and a block omega for Cl, V1, V2.

### Sample size calculation

A sample size of 184 patients in the ITT population, randomized in a 1:1 ratio into two treatment groups will provide at least 80% power for a two-sided t-test, at the significance level α = 0.05, to detect a difference of 0.5°C between the area under the curve 0–2 h (AUC_0–2)_ for patients receiving intravenous ibuprofen and those receiving acetaminophen. The sample size estimation was based on the assumption that the common standard deviation (SD) for the AUC_0–2_ was 1.2, an estimate based on data from a preliminary study conducted by the study sponsor.

A total of 200 patients (one hundred patients in the 6 month to 16 year age group, and another 100 patients in the less than 6 month age group) were to be randomized to ensure that 184 patients complete the study. Despite considerable effort, enrollment goals in the group less than the 6 months of age group were not met. Consequently the study was terminated with the enrollment of the planned 100 patients in the greater than 6 month age group.

## Results

**Fig. 1:**
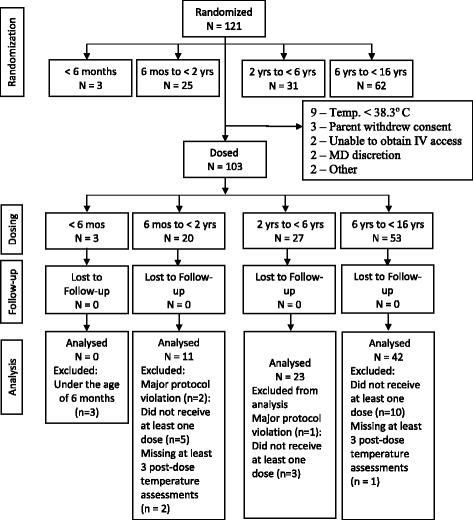
Patient Disposition. The patient deposition describes number of patients whom were randomized, dosed with study medication, lost to follow-up, and analyzed

A total of 121 patients were randomized into the study with 103 patients receiving a dose of study medication (Fig. 1). Nine subjects were excluded due to a temperature < 38.3°C prior to receiving the initial dose of study drug; three due to withdrawal of consent; two due to inability to obtain intravenous access, two at the medical doctors’ discretion, and two for unspecified reasons.

Baseline demographics were not different between the treatment groups (Table 2). The mean number of doses received was similar between the treatment groups (4 doses); however the median number of doses was one for those patients receiving acetaminophen and four for those receiving intravenous ibuprofen (Table 3). The duration of study drug was defined as the number of hours from the first dose to the last dose. Since 53% of subjects received only 1 dose of acetaminophen, these subjects have a duration of 0 h hence the median duration was 0.0 h. Patients receiving intravenous ibuprofen were more likely to receive multiple doses compared to patients receiving acetaminophen, 70% vs 47% respectively, while length of stay remained the same.

**Table 2:** Summary of demographic characteristics

	Acetaminophen	Intravenous Ibuprofen
Demographic	*N* = 53	*N* = 47
Age (years)		
Mean (SD)	6 (4.4)	7 (4.6)
*p*-value		0.098
6 months to < 2 years	14(26%)	6(13%)
2 to < 6 years	14 (26%)	13(28%)
6 to 16 years	25(47%)	28(60%)
Gender		
Male	26 (49%)	27 (57%)
Female	27 (51%)	20 (43%)
*p*-value		0.404
Race		
White	42 (79%)	42 (89%)
Black or African American	8 (15%)	5 (11%)
American Indian or Alaska Native	2 (4%)	0
Other	1 (2%)	0
*p*-value		0.346
Ethnicity		
Hispanic or Latino	24 (45%)	29 (62%)
Not Hispanic or Latino	29 (55%)	18 (38%)
*p*-value		0.102
Weight		
Mean (SD)	24.2 (15.2)	30.2 (19.5)
*p*-value		0.085

*N* number, *SD* standard deviation

**Table 3:** Summary of drug exposure

	Acetaminophen	Intravenous ibuprofen
	*N* = 53	*N* = 47
Total Number of Doses		
Mean (SD)	4 (3.8)	4 (3.7)
Median	1	4
Min, Max	1, 17	1, 23
1 Dose	28 (53%)	14 (30%)
2 Doses	3 (6%)	8 (17%)
3 Doses	2 (4%)	1 (2%)
4 Doses	1 (2%)	1 (2%)
5 Doses	1 (2%)	6 (13%)
6 Doses	9 (17%)	11 (23%)
> 6 Doses	9 (17%)	6 (13%)
Duration of Study Drug (hours)^a^		
Mean (SD)	15.4 (24.4)	16.6 (19.8)
Median	0.0	16.0
Min, Max	0.0, 117.5	0.0, 104.9

*N* number, *SD* standard deviation, *Min* Minimum, *Max* Maximum

^a^Duration of study drug is defined as the number of hours from the first dose to the last dose

**Fig. 2:**
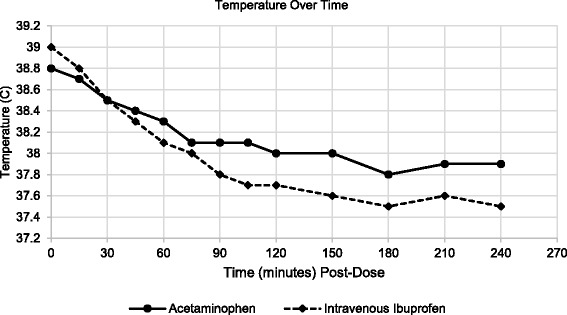
Temperature Over Time, 0–4 h Post-Dose. There was a significant difference between the two groups in the changes in temperature over time

**Fig. 3:**
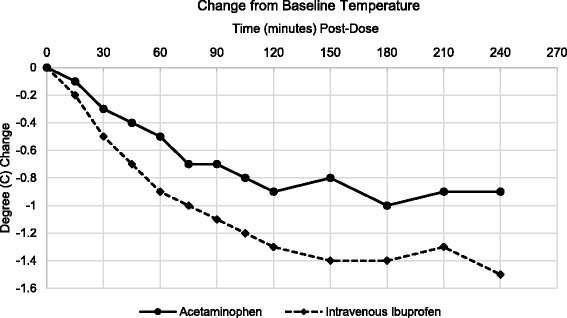
Change from Baseline Temperature over Time, 0–4 h Post-Dose. There was a greater reduction in temperature change from baseline temperature in the ibuprofen group compared to acetaminophen group

Treatment with intravenous ibuprofen compared to acetaminophen resulted in a significantly greater reduction in temperature as measured by the area under the change from baseline versus time curve (AUC_0–2_) (Table 4). Similar results were observed after four hours of treatment (Table 4). Secondary efficacy evaluations of changes in temperature over time demonstrate that treatment with intravenous ibuprofen resulted in a greater reduction in change from baseline temperature compared to treatment with acetaminophen. The difference between treatment groups was significant at 30 min after treatment and remained throughout the four hour period following dosing (Figs. 2 and 3).

**Table 4:** Area under the change from baseline versus time curve

AUC (0–2 h)	Acetaminophen	Intravenous Ibuprofen
N	50	46
Mean (SD)	-0.9 (0.89)	-1.5 (1.11)
Min, Max	-3.0, 0.7	-4.4, 0.1
*p*-value*		0.005
AUC (0–4 h)	Acetaminophen	Intravenous Ibuprofen
N	42	44
Mean (SD)	-2.6 (2.02)	-4.4 (2.59)
Min, Max	-7.4, 1.3	-12.0, 0.6
*p*-value*		<0.001
AUC (0–24 h)	Acetaminophen	Intravenous Ibuprofen
N	24	29
Mean (SD)	-26.6 (14.29)	-34.2 (17.97)
Min, Max	-66.1, 1.7	-72.9, 1.3
*p*-value*		0.099

*AUC* area under the curve, *Min* minimum, *Max* maximum, *N* number, *SD* standard deviation

*The analysis is based on an ANOVA model with fixed effects for treatment group

The *p*-value and 95% confidence interval are based on the difference in LS Means from the ANOVA model

After administration of a single dose, more patients who received intravenous ibuprofen reached an afebrile temperature (<38.3° C or 101.0° F) compared to patients who received acetaminophen (*p* = 0.036) (Table 5). The time to reaching an afebrile temperature was not significantly different between the two groups (mean was 2.2 h for ibuprofen as compared to 3.3 h for acetaminophen, *p* = 0.156) (Table 5).

**Table 5:** Summary of afebrile temperature

	Acetaminophen	Intravenous ibuprofen
Subjects Afebrile at 4 h Post-Dose		
Yes	40 (75%)	43 (91%)
No	11 (21%)	3 (6%)
*p*-value		0.036
Time to Afebrile Temperature (hrs)		
N	53	47
Number Censored	6 (11%)	2 (4%)
Mean (SE)	3.3 (0.67)	2.2 (0.47)
Median (95% CI)	1.5 (1.0, 2.5)	1.3 (1.0, 1.5)
25%–75%	1.0–4.0	0.8–2.1
*p*-value		0.156

*CI* confidence interval, *Hrs* hours, *N* number, *SD* standard deviation

Adverse events were reported for 54 of the 100 patients, with most (97%) being classified as mild to moderate in severity. There was no difference in the number of patients experiencing adverse events or the number of adverse events between treatment groups (Table 6). The most common adverse events were vomiting, infusion site pain (ibuprofen only), headache, nausea and diarrhea (Table 6). There were no deaths reported in this study. There were four (4%) subjects for whom six serious adverse events were reported. In the intravenous ibuprofen group, two subjects experienced four serious adverse events; one with pancreatitis and hepatitis and one with cardiac arrest and pneumothorax. In the acetaminophen group, two (2%) subjects experienced two serious adverse events; pleural effusion, and intra-abdominal abscess. None of the serious adverse events were deemed related to either intravenous ibuprofen or acetaminophen in the opinion of an independent data safety monitor.

**Table 6:** Adverse events occurring in ≥ 3 patients

	Acetaminophen, *N* = 53		Intravenous Ibuprofen, *N* = 47	
Adverse Event	*N* (%) Patients	# Events	*N* (%) Patients	# Events
Any AE^1^	26 (49%)	65	28 (60%)	66
Any AE, excluding infusion site reaction^2^	26 (49%)	65	24 (51%)	59
Infusion Site Pain	0	0	5 (11%)	7
Vomiting	3 (6%)	3	3 (6%)	4
Headache	1 (2%)	1	3 (6%)	3
Nausea	2 (4%)	2	3 (6%)	3
ALT Increased	1 (2%)	1	2 (4%)	2
AST Increased	2 (4%)	2	2 (4%)	2
Blood lactate dehydrogenase increased	3 (6%)	3	1 (2%)	1
Diarrhea	4 (8%)	4	1 (2%)	1
Hypokalemia	2 (4%)	2	1 (2%)	1
Pruritus	2 (4%)	2	1 (2%)	1

*N* number, *AE* adverse event, *ALT* alanine transaminase, *AST* aspartate transaminase

^1^ *p*-value = 0.321; ^2^ *p*-value >0.999

There were no differences between treatment groups in regards to the change from baseline to endpoint for pulse rate, respiratory rate, and systolic or diastolic blood pressures. Nor were there significant changes between treatment groups in regards to the change from baseline to endpoint for clinical chemistry, hematology or coagulation safety laboratory assessment.

Out of the three subjects under 6 months of age, a 5 and 3 month old received acetaminophen while a 1 month old received intravenous ibuprofen. The subject receiving ibuprofen had a 2.0° C temperature decrease while a 3 month old receiving acetaminophen had a 1.9° C decrease. The 5 month old receiving acetaminophen did not have temperature assessments. There were no safety concerns in the three patients.

**Fig. 4:**
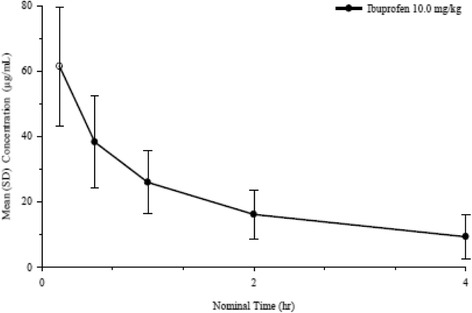
Mean concentration-time profile for intravenous ibuprofen

Plasma concentration values from patients receiving intravenous ibuprofen were used to calculate pharmacokinetics parameters (Table 7). In the 43 patients that received ibuprofen, the AUC from time zero to 4 h (AUC_0–4_) and maximum plasma concentration (C_max_) ranged from 22.96 to 162.0.6 microgram*hours/milliliter (mcg*hr/mL) and 15.91 to 96.31 microgram/milliliter (mcg/mL). The median time of observed maximum concentration was 10 min (Fig. 4), which corresponds to the end of the infusion. The elimination half-life was short and ranged from 0.79 to 2.87 with a mean of 1.55 h. The overall exposure increased with age. As expected, clearance and volume of distribution increased with age.

**Table 7:** Pharmacokinetic parameters for intravenous ibuprofen by age category

Age Category	N	AUC_0-t_ (mg · hr/mL)	AUC0-4 (mg · hr/mL)	Cmax (mg/mL)	Tmax (hr)	T1/2el (hr)	Cl (ml/h)	V_z_ (mL)
Birth to < 6 mo.	1	51.18	69.14	49.83	0.167	1.8	619.97	1053.72
6 mo. to < 2 years. (min, max)	5	71.15 (34.67, 95.20)	70.92 (34.67, 95.20)	59.24 (38.37, 92.02)	0.234 (0.167, 0.500)	1.78 (1.06, 2.35)	1172.5 (844.83, 1955.92)	2805.73 (2036.38, 3568.56)
2 yrs. to < 6 years. (min, max)	12	79.19 (19.00, 109.50)	80.25 (22.96, 123.58)	64.18 (15.91, 96.31)	0.309 (0.167, 0.767)	1.48 (0.79, 2.87)	1967.27 (1098.93, 4744.56)	3695.76 (1850.84, 5410.57)
6 years. to < 16 years. (min, max)	25	80.67 (40.00, 161.30)	85.73 (39.62, 162.03)	61.89 (31.03, 93.32)	0.212 (0.167, 667)	1.55 (0.79, 2.54)	4878.47 (998.60, 12536.67)	10314.21 (2638.76, 23964.19)

AUC0-t: Area under the concentration-time curve from time zero to the last measurable

concentration using linear-log trapezoidal rule

AUC0–4: Area under the concentration-time curve from time zero to 4 h

Cmax: Maximum observed concentration

Tmax: Time of observed Cmax

T½ el: Elimination half-life, calculated as ln(2)/Kel

Cl: Total body clearance, calculated as Dose/AUC0-inf

Vz: Volume of distribution, calculated as Dose/(Kel x AUC0-inf)

## Discussion

Fever, often the earliest and most visible signs of a disease process can increase the metabolic rate and exert harmful effects of the disease process [12]. Many healthcare providers agree that fever in children is usually not damaging and the significance of fever is often defined by its duration and the debilitating accompanying symptoms [1]. Fever needs to be treated with an antipyretic which is rapidly effective, well tolerated and in as much as possible, fulfills the highest requirement of safety [1].

Oral ibuprofen and acetaminophen are the most commonly used antipyretics in the hospital setting. One systematic review found that both these medications have similar tolerability and safety profiles in terms of gastrointestinal symptoms, asthma and renal adverse effects [13]. Aspirin, though highly effective, is contraindicated due to its association with Reye’s syndrome [1]. However, hospitalized patients with endotracheal intubation, sedation, reduced level of consciousness, reduced gastric motility, nausea, and vomiting, or a recent or intraoperative procedure make oral or rectal antipyretic preparations undesirable due to their inability to administer or unpredictability of the absorption [7]. In addition, as the drug may not be equally distributed throughout the suppository, splitting a suppository may not provide a predictable or reliable dose. Additionally, different rectal preparations have substantially different absorption characteristics causing variations in bioavailability [4, 7]. Intravenous antipyretic, given their route of administration and path of absorption have proven superior to oral antipyretics at reducing fever rapidly.

This randomized, open-label study evaluated the efficacy, safety, and pharmacokinetics of intravenous ibuprofen in febrile hospitalized pediatric patients. In this population of hospitalized pediatric patients, intravenous ibuprofen was more effective than acetaminophen in reducing temperature after a single dose of either medication.

Overall, the most common adverse event reported during the study was vomiting, while the most common adverse event reported in the ibuprofen treated patients was infusion site pain. Serial measurement of chemistry, hematology, and coagulopathy indicate that for the duration of exposure to intravenous ibuprofen, there were no clinically significant renal, gastrointestinal, or bleeding effects of intravenous ibuprofen in the 120 h after dosing or 1 week following study initiation.

The clearance and volume of distribution increased with age which was expected when taking into consideration the common-size related change in clearance for small chemical entities. In addition, the mean plasma concentration–time pattern and the pattern of maximum observed concentration and time of observed C_max_ are consistent with the values found in adult patients treated with intravenous ibuprofen (Cumberland Pharmaceuticals Inc., unpublished).

Findings from this study are also consistent with the growing body of knowledge supporting the use of ibuprofen as a treatment in the pediatric population. Perrott et. al. [14] conducted a meta-analysis of randomized controlled trial of ibuprofen and acetaminophen for pediatric pain and fever. In this review of over 127 studies, 17 studies met their inclusion criteria and provided 10 data sets for fever reduction and 17 data sets for safety analysis. Half of the fever studies reported efficacy in terms of the mean between drug differences in temperature at 2, 4, and 6 h after treatment while the other half reported efficacy in terms of mean between drug difference in temperature reduction from baseline at 2, 4, and 6 h. In the context of single doses of ibuprofen, 5 to 10 mg/kg was the superior antipyretic when compared to 10 to 15 mg/kg of acetaminophen. The superiority of ibuprofen as an antipyretic was more pronounced at 4 and 6 h after treatment with effect sizes in the order of 0.30. Following this metric, approximately 15% more children were likely to have fever reduction at 4 and 6 h with ibuprofen as compared to acetaminophen. Restricting the analysis to 10 mg/kg of ibuprofen compared to 10 to 15 mg/kg of acetaminophen, roughly double the effect size in favor of ibuprofen which corresponds to a 38% increase in the number of children likely to have a reduce fever at 4 h following treatment [14]. With regards to safety, the data suggests that there was no evidence that either that treatment with either ibuprofen or acetaminophen are less safe than each other or placebo [14]. These findings were also consistent with another meta-analysis that concluded that ibuprofen was a more efficacious antipyretic in fifteen studies and no significant difference in an additional fifteen studies [15]. Safety findings in these meta-analyses also remained consistent with the safety profiles established in previous studies found in the literature [16–18]. One large scale randomized trial conducted by Lesko and Mitchell found in 84,192 febrile children that a single dose or short-term repeated doses of 12 mg/kg acetaminophen versus 5–10mg/kg ibuprofen, that safety did not differ according to drug [17].

Limitations of this study involved the selection of the comparator drug. At the onset of this study, intravenous ibuprofen, was the only FDA approved antipyretic with an intravenous route of administration. While an IV form of acetaminophen is now commercially available (approved in 2010), it was not FDA approved or commercially available in the United States when the study was designed, initiated, or during early enrollment.

The use of oral or rectal acetaminophen as the comparator was not optimal given that at times absorption and utilization of oral or rectal preparations of medication can be unpredictable and lead to sub-therapeutic level of the medication. In practice, many pediatricians utilize acetaminophen at a dose of 15 mg/kg of body weight. Recommended acetaminophen dosing varies from 7.5 mg/kg every 6 h (for children ≤ 10 kg) to 15 mg/kg every 4 h (for children > 33 kg) to 12.5mg/kg every 4 h or 15mg/kg every 6 h for children ≥ 2 years of age (maximum daily dose of 75 mg/kg/day) [19, 20]. The study was designed to allow for multiple doses at a frequency of every 4 h. For the acetaminophen dose, 10 mg/kg was chosen to stay consistent with acetaminophen recommendations and to limit potential renal and hepatic toxicities should repeated dosing of acetaminophen be administered during the treatment period.

Enrollment in the ibuprofen treatment arm was larger in the 6–16 years age group (ibuprofen – 57%, acetaminophen - 48%) when compared to acetaminophen than in the other age groups (6 months -2 years [ibuprofen -17%, acetaminophen 25%] and 2 years – 6 years [ibuprofen – 26%, acetaminophen – 27%]). Given that difference disease processes cause fever in infants compared to older children and adolescents and the different mean weight of the subjects in the groups, there potentially could be different pharmacokinetic responses based on these variables.

Other limitation of the study included the relatively small number of patients studied in children under the age of 6 months and a relatively short duration of exposure. The duration of exposure to ibuprofen is, however, consistent with the recommendation that intravenous ibuprofen should be at the lowest effective dose and the shortest duration possible [5]. Additional research is needed in the less than 6 months age population to determine the safety and efficacy of intravenous ibuprofen in that age population.

## Conclusions

This study demonstrated that a single 10 mg/kg dose of intravenous ibuprofen provided a significant reduction of temperature for febrile pediatric patients compared to those that received 10 mg/kg acetaminophen. The reduction in temperature was significant at 2 h and 4 h post-treatment and a treatment effect was demonstrated over 24 h. There were no clinically significant differences in adverse events, including renal function, bleeding, or gastrointestinal events between the treatment groups throughout the study. There were no clinically significant differences in laboratory values between the treatment groups or serious adverse events. Intravenous ibuprofen is an effective option for the reduction of fever in hospitalized pediatric patients.

## Additional file

Additional file 1: Participating sites and their corresponding institutional review boards. (PDF 18 kb)

## Abbreviations

AEs: Adverse events
ALT: Alanine transaminase
ANCOVA: Analysis of covariance
ANOVA: Analysis of variance
APAP: Acetaminophen
AST: Aspartate transaminase
AUC: Area under the curve
C: Celsius
CI: Confidence interval
CL: Total body clearance
CTM: Clinical trial material
DSM: Data safety monitor
EEP: Efficacy-evaluable population
F: Fahrenheit
FDA: Food and drug administration
IBU: Ibuprofen
IRB: Institutional review board
ITT: Intent-to-treat
IV: Intravenous
Kg: Kilogram
Max: Maximum
Mg: Milligram
Min: Minimum
MOS: Months
N: Number
PK: Pharmacokinetic
PRN: As needed
SD: Standard deviation
T12/el: Elimination half-life
Tmax: Time of observed Cmax
V: Volume of distribution, Wt, Weight
Vz: Volume of distribution
YRS: Years
