# Supplementary material for: A multicenter, randomized, open-label, active-comparator trial to determine the efficacy, safety, and pharmacokinetics of intravenous ibuprofen for treatment of fever in hospitalized pediatric patients
Source: BMC Pediatr. 2017 Feb 1;17:42. doi: 10.1186/s12887-017-0795-y (PMC5286864; doi:10.1186/s12887-017-0795-y)
Supplement: Additional file 1: — Participating sites and their corresponding institutional review boards. (PDF 18 kb) [file 12887_2017_795_MOESM1_ESM.pdf]

Sites and their Corresponding IRB's

Site Name	CORRESPONDING IRB/IEC
Baptist Medical Center - South	Baptist Health Institutional Review Committee
The Joseph M. Still Burn Center	Western Institutional Review Board
Maricopa Medical Center	Maricopa Integrated Health System, Institutional Review Board
Louisiana State University Health Science Center -Shreveport	Louisiana State University Health Science Center –Shreveport, Institution Review Board
Kosair Children's Hospital	The University of Louisville Institutional Review Board
Baptist Medical Center - East	Baptist Health Institutional Review Committee
Howard University	Howard University Institution Review Board
Children's Memorial Hermann Hospital	UTHSC at Houston, The Committee for the Protection of Human Subjects
Children's Hospital at St. Francis	Saint Francis Health System, General Studies Institutional Research Ethics Board
Ochsner Clinic Foundation	Ochsner Clinic Foundation, Institutional Review Board
Children's Hospital at Michigan	Wayne State University, Human Investigation Committee
The Danbury Hospital	The Danbury Hospital Institutional Review Board
Staten Island University Hospital	Staten Island University Hospital, Institutional Review Board
LeBonheur Children's Hospital	The University of Tennessee Health Science Center Institutional Review Board
Loma Linda Univ. Children's Hospital	Loma Linda University Sponsored Research, Institutional Review Board
Jackson Memorial Hospital, University of Miami	University of Miami, Human Subject Research Office
Florida Hospital	Florida Hospital, Institutional Review Board
Duke University	Duke University Health System IRB Office
Texas Children's Hospital	Institutional Review Board for Baylor College of Medicine and Affiliated Hospitals
Children's Hospital of Orange County	CHOC Industry Track Institutional Review Board
Children's Medical Center - Dallas	University of Texas Southwestern Medical Center, Institutional Review Board
